# MolOrgGPT: *De Novo* Generation via Large Language Models and Reinforcement Learning

**DOI:** 10.1021/acs.jcim.5c02400

**Published:** 2026-01-07

**Authors:** Pablo Varas Pardo, Oscar Toledano, Guillermo Marcos-Ayuso, David Quesada, Nuria E. Campillo

**Affiliations:** † AItenea Biotech S.L., 28014 Madrid, Spain; ‡ Instituto de Ciencias Matemáticas (ICMAT-CSIC), 28049 Madrid, Spain; § 16722 Universidad Autónoma de Madrid, Escuela de Doctorado, 28049 Madrid, Spain; ∥ Centro de Investigaciones Biológicas Margarita Salas (CIB Margarita Salas−CSIC), 28040 Madrid, Spain

## Abstract

We present a general framework for the *de novo* design of small molecules with desirable chemical properties, developed to aid the creation of novel chemical entities with potential therapeutic use. The system is built upon a foundational Large Language Model trained on a large comprehensive chemical database capable of generating structurally diverse and synthetically accessible compounds. It is then fine-tuned through reinforcement learning to enhance its capacity to generate molecules tailored to specific biological targets. As a case study, we apply this framework to design molecules targeting key proteins involved in Alzheimer’s disease. The generated compounds underwent molecular docking studies to assess their binding affinities and prioritize candidates with optimal predicted interactions. The top-ranked molecules were further analyzed based on their binding modes and key molecular interactions with the target proteins. The results suggest that our generative model produces viable, drug-like molecules with favorable interactions, underscoring its potential as a valuable tool in early stage drug discovery.

## Graphical abstract

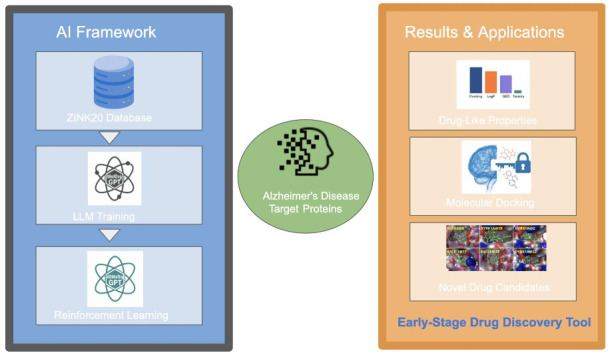

## Introduction

Recent advances in artificial intelligence (AI) have radically changed drug discovery.^1–4^ Given the immensity of the chemical space,^5,6^ AI tools and methods have become essential for exploring such space, generating novel molecules that interact with relevant protein pockets,^7^ and identifying patterns in molecular data to optimize the desired pharmacological properties,^8^ to name but a few uses.

Three major approaches exist to address the challenge of designing new molecules that effectively bind to specific protein pockets.^9^ The first one, the *ligand-based* approach, starts with a set of active molecules from which the main features (substructures responsible for activity) are extracted. Once obtained, these features are combined to create new molecules.^10–12^ The effectiveness of this approach has been extensively proven experimentally.^13–15^ However, its major problem is that a database of active molecules for a given protein is not always available; this inherently limits the exploration of chemical space. The second approach is *structure-based*.^16,17^ One starts with the protein pocket of interest, which is input into the model that then designs new molecules based on such protein. The main issue with this approach is its tendency to generate unrealistic molecules,^18,19^ typically neglecting crucial aspects, such as their nontoxicity or drug-likeness. As a consequence, while the generated molecules may initially appear promising, most of them ultimately fail to meet the criteria necessary to qualify as viable drug candidates.^20^ Although this approach has gained popularity in recent years and already provided successful cases,^21^ it still needs to be validated through biochemical assays.^22^ Finally, the third approach treats the problem as an *optimization* task, where, using reinforcement learning (RL), an agent learns to produce molecules that bind with high affinity to a protein pocket.^23^ With the availability of docking tools,^24–26^ which simulate the binding affinity between a molecule and a given protein, the model can learn to produce high-affinity molecules.^27–30^ This approach can overcome the inherent limitations of the other two methods and address additional challenges, such as optimizing around a predefined scaffold or designing multitarget compounds capable of effectively interacting with multiple protein pockets.^31–33^

Large Language Models (LLMs), built upon the Transformer architecture, have shown exceptional capabilities in capturing complex dependencies within sequences in multiple domains through their built-in attention mechanism.^34^ In particular, in our domain of molecular generation, this makes them highly suitable for both predicting molecular properties and generating novel molecules, as^35,36^ reflect. RL, especially the Proximal Policy Optimization (PPO) algorithm,^37^ has become essential in the fine-tuning of LLMs. In drug discovery, RL can guide the model to produce molecules with optimal properties of interest, significantly enhancing the chances of developing effective drug candidates.^38,39^

This paper introduces MolOrgGPT, a family of three foundational models for molecular generation. We develop three variants of a Generative Pretrained Transformer (GPT-2)^40^ trained on 800 M molecules from the ZINC20 database,^41^ one of the most comprehensive data sets available for molecular LLM training. To our knowledge, this represents the largest chemical data set and among the largest generative models trained so far for *de novo* molecular design. Furthermore, using RL, we develop ADMolOrgGPT models specifically designed to generate novel molecules targeting protein pockets associated with Alzheimer’s Disease (AD). This approach demonstrates the potential of GPT models to capture patterns in chemical language that enable the optimization of docking scores against a protein pocket, something that has rarely been explored due to the high computational cost of docking and the challenges of integrating it within generative pipelines.

Our strategy consists of two main stages:

1. *Pretrain decoder-only transformer models.* In this stage, we developed MolOrgGPT-M, L, and XL, based on the GPT-2 architecture, using a huge subset from the ZINC20 database.^41^ Its molecules were available in the popular Simplified Molecular Input Line Entry System (SMILES)^42^ format, but as Section *Less Successful Attempts* explains, we finally adopted a variation of this representation called Self-Referencing Embedded Strings (SELFIES),^43^ which ensures the chemical validity of all generated molecules through a set of predefined rules preventing valence violation,^44^ with the following desirable outcomes: increasing the chemical diversity of generated molecules, providing richer latent representations for molecular property prediction, and delivering foundational models for drug discovery task-specific fine-tuning.
2. *Reinforcement Learning for guided molecular generation.* Building upon our pretrained MolOrgGPT models, we employed a PPO-RL algorithm to guide molecular generation. Given our interest in neurodegenerative diseases, we focused on generating molecules targeting three protein pockets known to be associated with AD: Dual-Specificity Tyrosine-phosphorylation-Regulated Kinase 1A (DYRK1A),^45^ β-Secretase 1 (BACE-1),^46^ and Butyrylcholinesterase (BuChE).^47^

We compare the performance of our approach against two baseline models: Pocket2Mol^48^ and DrugGPT,^49^ which respectively use a pocket-based and a ligand-based condition approach. Our results suggest that ADMolOrgGPT stands out in generating molecules with desired properties. Additionally, our model excels in scaffold-constrained generation, learning to produce high-affinity molecules for a protein pocket based on a predefined scaffold. Furthermore, the flexibility of the PPO reward function allows us to generate molecules simultaneously optimized for multiple targets, an innovative capability in the context of pocket-based drug design. To further assess and confirm the binding capabilities of the designed compounds, molecular docking simulations were undertaken with the highest-scoring molecules produced by the generative model.

## Methods

### MolOrgGPT: Foundational LLMs for Molecular Generation

Transformers^50^ constitute a well-known deep neural network architecture with three main variants: encoder-only, decoder-only, and encoder–decoder models. In the field of molecular generation, many existing approaches have utilized an encoder to extract features from a protein pocket, followed by a decoder that generates candidate molecules.^7,49,51^ However, these methods frequently encounter challenges due to the limited availability of protein–ligand affinity data and the difficulty of integrating RL to fine-tune relevant properties of the generated molecules. Examples of encoder-decoder models include TamGen^52^ and Lingo3dMol.^7^

Encoder-only models include Bidirectional Encoder Representations from Transformers (BERT),^53^ the Robustly Optimized BERT Pretraining Approach (RoBERTa),^54^ and chemical domain-specific models available at Hugging Face, including Roberta-Zinc^55^ and ChemBERTa.^52^ These models are designed to process input sequences bidirectionally, making them effective for tasks such as classification and representation learning of molecules.

Conversely, decoder-only models, such as MolGPT^56^ or DrugGPT,^49^ have shown promising results in molecular generation tasks. Crucially, the decoder-only architecture itself can be integrated into RL workflow for optimization, motivating its choice in our design.

### Model Architecture

We employ a decoder-only Transformer architecture based on Karpathy’s nanoGPT codebase.^57^ Each input symbol, a SELFIES token, is mapped into an embedding vector and combined with positional encoding to retain token order information. These representations flow through a stack of Transformer blocks. Each block contains a multihead self-attention layer that allows every token to attend to all preceding tokens, followed by a position-wise feed-forward network that captures complex feature interactions. Finally, a linear layer converts the last hidden state at each position into a probability distribution over the vocabulary to predict the next token. To keep attention focused, we include two complementary masks:

1. *Look-ahead mask*, preventing the model from attending to future tokens.
2. *Padding mask*, facilitating ignoring tokens added to equalize sequence length, represented with the [PAD] token, ensuring they do not influence the chemical tokens.

MolOrgGPT-M, L, and XL share the overall architecture described above, but as seen in Table 1, differ in the number of transformer blocks (Layers), the dimensionality of the initial embedding layer (*d*_model_) and the number of attention heads (Heads).

**Table 1:** Architecture Hyperparameters for MolOrgGPT Variants

model	parameters	layers	*d*_model_	heads
MolOrgGPT-M	302M	24	1024	16
MolOrgGPT-L	702M	36	1280	20
MolOrgGPT-XL	1474M	48	1600	25

For computational convenience, the output layer dimension was set to 512 (a power of two, which aligns with common GPU memory-alignment and parallelization optimizations).^58^ Further details regarding these design choices are available in Section Training process.

### Data Set

We utilized a subset of the ZINC20 ^41^ database, containing approximately 800 M molecules in SMILES format with molecular weights ranging from 250 to 500 Da and a partition coefficient (LogP) between 1 and 4.5. This selection aimed to train a molecular generation model that adheres to the well-known Lipinski’s rule of five,^59^ to ensure that the resulting molecules possess physicochemical properties suitable for drug discovery.

### Training Process

The first step in training LLMs is typically a tokenization process. Choosing an appropriate tokenizer is critical, since LLM outputs vary considerably depending on this choice. We considered three tokenizers:

- *Character-level SMILES tokenizer*,^60^ splitting a SMILES string into individual characters (resembling Markov’s approach when he introduced Markov chains).
- *Byte-pair-encoding (BPE) SMILES*,^61^ which merge common character sequences into grouped tokens. This is widely used in natural language processing.
- *Bracket-level SELFIES tokenizer.* ^62^ This representation uses SELFIES, a robust alternative to SMILES, splitting a SELFIES string at each closing bracket so that every token corresponds to a complete bracketed SELFIES symbol.

After training the LLMs using these different tokenizers, we concluded that the bracket-level SELFIES one was the most appropriate choice for our purposes: a key advantage of SELFIES was that it consistently generates syntactically valid molecules, something of major relevance for our subsequent RL stage. Further details on our experiments with the other two tokenizers can be found in the section Less Successful Attempts.

To tokenize the molecules in our data set, we added three special symbols: [PAD], [SOS], and [EOS] (see Table 2), denoting padding, start of sequence, and end of sequence, respectively.

**Table 2:** Vocabulary Composition

category	example	purpose
Special	[SOS]	Sequence control
Branch (ramifications)	[Branch1]	Open branch X positions back
Ring (ring closures)	[Ring2]	Close ring X positions back
Pure atoms	[=C]	Add atom with bond order
Stereochemistry (chiral centers)	[C@@H1]	Chiral center orientation
Directed bond (“/” or “\”)	[/C]	Bond direction
Formal charge/explicit H	[NH1]	Charge or explicit H

Figure 1 illustrates the tokenization pipeline for a single molecule: the SMILES string is converted to SELFIES and then tokenized into model input tokens. Once the 800 M molecules were converted into SELFIES and tokenized, we filtered the data set to retain only those molecules containing at most 82 tokens, including [SOS] and [EOS] tokens, since such threshold allowed us to cover 97.5% of the entire data set. Molecules shorter than 82 were padded with the [PAD] token.

**Figure 1:**
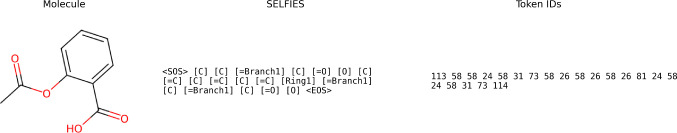
Tokenization pipeline example.

We then trained three separate models (MolOrgGPT-M, MolOrgGPT-L, and MolOrgGPT-XL) using an Adam optimizer with a learning-rate scheduler. For computational reasons, the models were trained using stochastic sampling over a subset of the tokenized data instead of a full pass through the entire corpus, with early stopping when the training loss converged. Given that each effective batch per iteration contains 519168 tokens and the training allowed up to 10000 iterations, the maximum number of distinct molecules that could be sampled during pretraining was approximately 65M.

### ADMolOrgGPTs: Guiding Molecular Generation Targeting Alzheimer’s Disease through RL

Once the foundational MolOrgGPT models were available, we applied RL to improve their generation capabilities so as to deliver molecules with good properties as desired, in our case, generating molecules that are more likely to dock with AD recognized protein pockets. The effectiveness of this strategy will be demonstrated in Results.

### RL Algorithm

We used PPO^37^ to implement RL. It is a widely adopted method that aims to maximize expected reward while ensuring that policy updates do not lead to drastic changes, which can destabilize training.

### Reward Modeling

Reward modeling is a key step when applying RL. In our case, to guide the generation process we used normalized reward functions based on the logistic one, mapping input values to the interval [0,1]. This guarantees numerical stability as well as provides smooth gradients for PPO. By rescaling the input range of the logistic function, we control the steepness of the transition from low to high reward values, effectively tuning how sharply the model differentiates between poor and good docking scores.

### Single-Pocket Reward

For the single-pocket formulation, used in Sections Single Pocket-Based Molecular Generation and Scaffold-Constrained Pocket-Based Generation, we linearly rescaled the docking empirical range [−12, – 6] kcal/mol, provided by Vina,^24^ to [−6, 6]. This rescaling allows that the docking score *x*, once mapped into this interval, is passed through the logistic function (eq 1), ensuring a clearer separation between weak and strong binders,

1 $$s_{\mathrm{dock}}\left(x\right)=\frac{1}{1+e^{-x}}$$

finally transformed into the reward (eq 2):

2 $$R_{\mathrm{dock}}\left(x\right)=1-s_{\mathrm{dock}}\left(x\right)$$

so that higher *R*_dock_ means better (more negative) docking.

### Two-Pocket Reward

The two-pocket reward variant, employed in Section Two Pocket-Based Molecular Generation, linearly aggregates the docking scores *x*_1_ and *x*_2_ of the two protein pockets following eq 3:

3 $$R_{\mathrm{dock}}^{\left(2\right)}\left(x_{1},x_{2}\right)=\alpha \left[1-s_{\mathrm{dock}}\left(x_{1}\right)\right]+\beta \left[1-s_{\mathrm{dock}}\left(x_{2}\right)\right],,\alpha +\beta =1,,\alpha ,\beta \geq 0$$

As is standard when aggregating objectives in multiobjective RL,^63^ we reflect a similar preference between both pockets by setting α = β = 0.5 in Section Two Pocket-Based Molecular Generation.

### Computational Cost

The large-scale training phase was carried out leveraging eight NVIDIA GPUs, each equipped with 40 GB of high-bandwidth memory for 10000 iterations. The total times for training the models are reflected in Table 3:

**Table 3:** Time Required for Pretraining Models

model	time (h)
M	40.85
L	71.32
XL	95.98

The RL stage requires repeated docking evaluations and forward passes through the model. Because the computational load depends on model size, RL was run using 1 GPU for models M and L, and 2 GPUs for the XL model. For all models, the time per RL epoch remained stable at approximately 27 min. In general, the XL model converged faster to an optimal solution, but as a trade-off, it required more computation time during pretraining.

### Docking Score Benchmarking

After applying the RL workflow, the docking scores of the proposed molecules showing highest score values were obtained using the Schrödinger suite^64^ to test their validity and analyze the interactions established between protein and ligand. They were also compared with the docking scores of reference molecules which are known to effectively bind to DYRK1A,^65^ BuChE,^66^ and BACE-1.^67^

To conduct the docking analysis and determine the most favorable binding poses at the target site, in a first stage, the SMILES codes generated by the LLM model were transformed into three-dimensional structures using the LigPrep utility embedded in the Schrödinger suite,^68^ which uses the Epik module^69^ to generate the feasible ionization states at physiological pH conditions. Energy minimization was performed to refine these molecular structures, employing the OPLS4 force field.^70^ On the other hand, receptor proteins were also prepared, using the Protein Preparation Workflow,^71^ which includes the assignment of the bond order, the assignment of the protonation state according to physiological conditions, hydrogen bond formation and a final structural optimization using OPLS4 force field. The grid for generating the docking poses of the proposed ligands was centered at the position of the ligand in the reference crystalline structure (5TOL, 6EIF, and 7Q1P protein structures for the BACE-1, DYRK1A, and BuChE proteins, respectively). Finally, relevant ligand–protein interactions of the resulting poses with highest docking scores were also analyzed.

## Results

The following subsections present the results attained with the ADMolOrgGPT-XL model for various generation tasks to enhance clarity. As the largest model, it generally outperformed the other two models. A comprehensive comparison of performance according to model size is in Section Model Comparison According to Size. The best molecules generated with different strategies are reported in Tables S1–S6 of the Supporting Information.

### Single Pocket-Based Molecular Generation

We evaluate the performance of the proposed models in generating molecules against the three proposed protein pockets. In addition, we compare their results with those of Pocket2Mol and DrugGPT, two widely used benchmark models with publicly available code. To ensure fair comparison, we adopt standardized assessment protocols.^72^

Each model will be assessed according to nine complementary criteria relevant in drug development:

- *Vina Docking Score (kcal/mol).* The binding affinity calculated with AutoDock Vina^24^ through the AutoGrow4 workflow.^73^
- *Quantitative Estimate of Drug-Likeness (QED).* ^74^ A score ranging from 0 to 1, calculated using RDKit’s QED module.^75^
- *LogP.* The compound’s lipophilicity obtained with RDKit Descriptors MolLogP.^75^
- *Synthetic-Accessibility Score (SAS).* ^76^ It measures how easily a molecule can be synthesized, with scores ranging from 1 (easy) to 10 (difficult). It is computed using the RDKit contrib script sascorer.^75^
- *Molecular Diversity.* Defined as (1−S̅) , where S̅ is the mean pairwise Tanimoto similarity between 2048-bit Extended Connectivity Fingerprints (ECFP4)^77^ of molecules.
- *Toxicity.* Assessed using Chemprop,^78^ a message-passing neural network that predicts the probability of toxicity across 12 Tox21 end points.^79^ A molecule passes the toxicity filter if its predicted probability is below 0.5 for all 12 end points.
- *Validity, Uniqueness, and Novelty.* Measured following the GuacaMol benchmark:^80^ validity is the fraction of syntactically valid SMILES; uniqueness is the proportion of nonduplicate valid molecules; novelty is the proportion of generated unique molecules absent from the reference database.

Broadly speaking the desired properties of the generated molecules should include: a high binding affinity, reflected through a low Vina docking score; high drug-likeness, indicated by a high QED value; a LogP within the optimal range of 0 to 5; a low SAS score, suggesting ease of synthesis; high molecular diversity; and nontoxicity across all 12 Tox21 end points.

Table 4 presents the results for a single generation batch of 256 molecules per protein pocket for the three models under evaluation. The results suggest that our model outperforms the baselines on several key properties, while remaining comparable in the other ones. ADMolOrgGPT achieves slightly lower docking scores, while remains comparable to the two baselines. This is significant considering that both Pocket2Mol and DrugGPT were trained on the CrossDocked^81^ and jglaser/binding_affinity^82^ data sets respectively, which consist solely of protein–ligand complexes, whereas our model was trained only on ZINC20 and optimized via RL. In turn, our model surpasses both baselines in terms of nontoxicity, QED, and percentage of molecules with LogP between 0 and 5. Lastly, although all models show comparable diversity, ADMolOrgGPT and Pocket2Mol achieve the highest validity, uniqueness, and novelty in generation. In Section Analysis of Baseline Training Data Sets we discuss the inherent generative bias of the models due to the different data sets used for training and its implications in the docking score and the physicochemical properties of the generated molecules.

**Table 4:** Model Evaluation across Molecular Properties^a^

	Pocket2Mol	DrugGPT	ADMolOrgGPT
Vina Score (kcal/mol, ↓)	–8.60 ± 1.33	–8.81 ± 1.21	–8.29 ± 1.22
QED (↑)	0.64 ± 0.16	0.59 ± 0.18	0.68 ± 0.16
LogP (%, 0–5, ↑)	86.72 ± 1.23	82.42 ± 1.37	92.97 ± 0.92
SAS (↓)	3.37 ± 0.95	3.26 ± 0.98	3.31 ± 1.02
Diversity (↑)	0.87 ± 0.05	0.86 ± 0.05	0.86 ± 0.04
Toxicity (%, ↓)	36.07 ± 1.73	29.04 ± 1.64	8.59 ± 1.01
Validity (%, ↑)	99.09 ± 0.34	100.00 ± 0.00	100.00 ± 0.00
Uniqueness (%, ↑)	98.96 ± 0.37	100.00 ± 0.00	100.00 ± 0.00
Novelty (%, ↑)	98.44 ± 0.45	99.61 ± 0.23	100.00 ± 0.00

a ↑ Higher is better. ↓ Lower is better.

### Scaffold-Constrained Pocket-Based Generation

Scaffolds in drug development are essential molecular structures that serve as building blocks for designing new drugs.^83–86^ Consequently, the ability to create new molecules starting from a scaffold as a foundation is highly important. As an example, we selected the scaffold in Figure 2. According to our internal group studies, molecules that contain this substructure have demonstrated favorable docking scores in Vina simulations for the DYRK1A protein pocket. This section assesses ADMolOrgGPT’s ability to optimize molecular generation based on a specific protein pocket while maintaining a given scaffold, which we designate *as scaffold-constrained molecular generation*.

**Figure 2:**
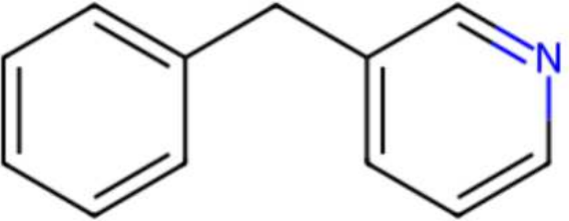
Scaffold selected for constrained pocket-based generation

For this, we trained the model under scaffold constraints and evaluated docking improvements across epochs. This experimental setup allows us to directly assess how optimization proceeds under fixed structural cores. Results are presented in Figure 3, which shows the docking scores attained with our constrained two-stage process, under constrains. The blue boxplot reflects the initial scores, whereas the orange one corresponds to those obtained after optimization. Note that just after five training epochs, the model learned to generate molecules that maintain the scaffold while considerably improving the docking score. Compared to the molecules produced by ADMolOrgGPT for the DYRK1A protein in the experiment described in Section Single Pocket-Based Molecular Generation, the mean docking score improved from −8.58 to −9.43, suggesting an important performance improvement. This outcome highlights the effectiveness of our approach, where a predefined scaffold is identified and then optimized to generate molecules with better docking scores.

**Figure 3:**
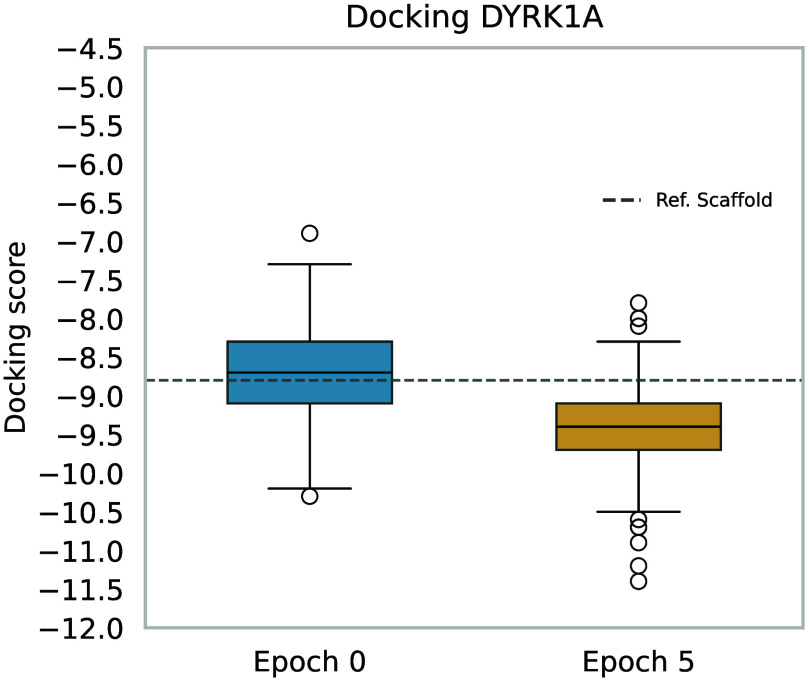
Scaffold-constrained docking optimization.

A key aspect of our methodology is the ability to track policy shifts in the LLM, allowing us to extract mechanistic insights from the RL process. As shown in eq 4, the Vina scoring function depends on Gaussian terms, hydrophobic contributions, repulsion interactions, hydrogen bonding, and ligand flexibility:

4 $$E_{\mathrm{Vina}}=-0.0356\mathrm{Gauss1}-0.0052\mathrm{Gauss2}+0.8402\mathrm{Repulsion}-0.0351\mathrm{Hydrophobic}-0.5874\mathrm{Hbonding}+0.0585N_{\mathrm{rot}}$$

Figure 4 shows how the probability of selected tokens evolves from epoch 0 to epoch 5 as the model improves the Vina docking score of the generated molecules. The DYRK1A pocket is predominantly hydrophobic and aromatic, with few relevant H-bond donors. Consequently, the loss of H-bond sites ([N] *↓*, [O] *↓*) has limited impact, whereas increases in rigidity and aromaticity ([Ring1] *↑*, [=C] *↑*, [=N] *↑*) consistently enhance packing and Gaussian terms. This drives the RL process toward more rigid, compact, and aromatic structures, which naturally lead to achieving better docking scores.

**Figure 4:**
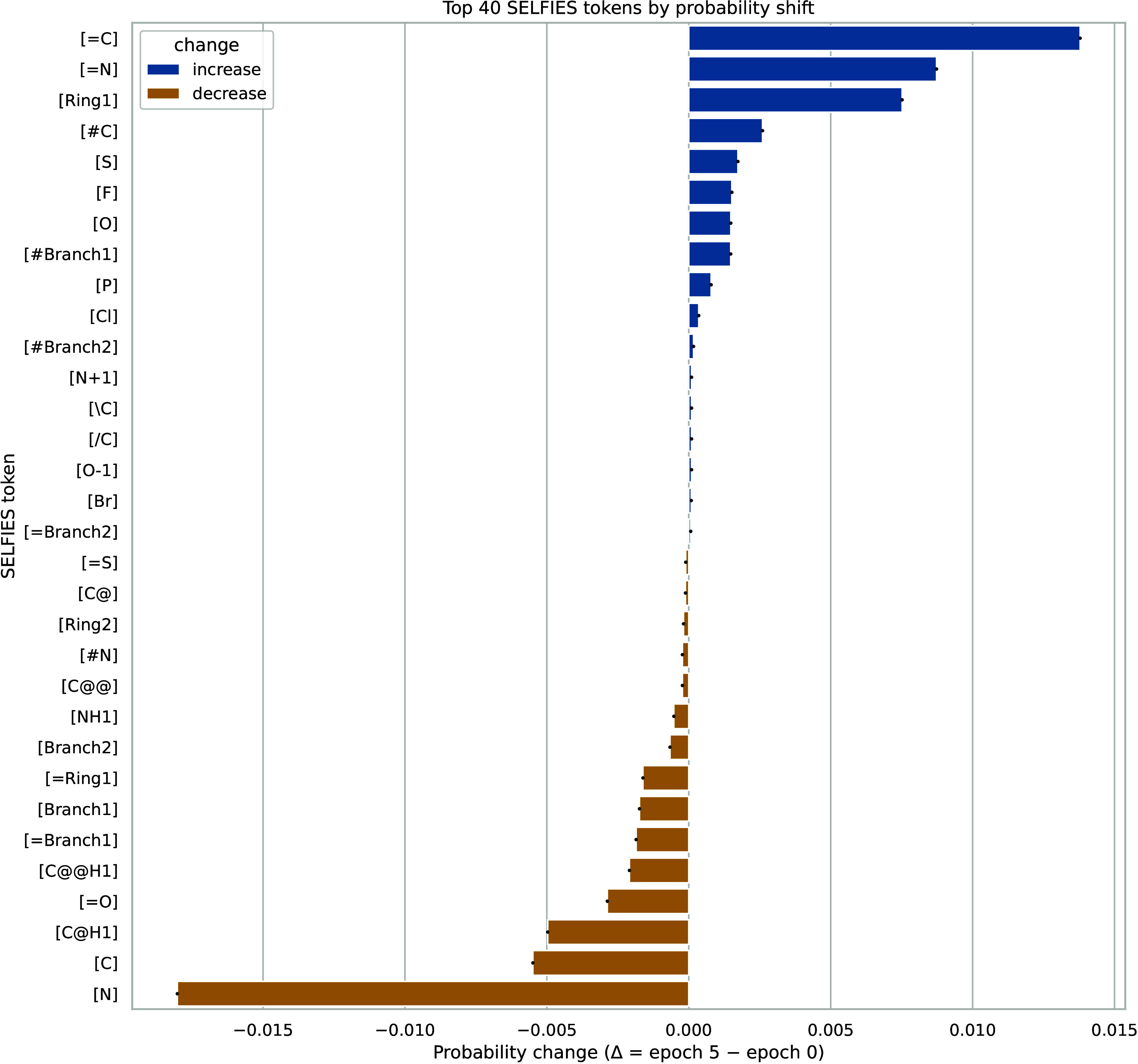
Token probability evolution across epochs.

### Two Pocket-Based Molecular Generation

A significant challenge and opportunity in modern drug discovery lies in designing molecules that effectively target multiple protein pockets,^87,88^ with current state-of-the-art models for pocket-based generation are being typically limited to a single protein pocket as input. Our literature review indicates that LigBuilder V3 ^89^ utilizes a genetic algorithm to generate molecules targeting multiple protein pockets. However, we were unable to reproduce its code for experimental comparisons.

Here we assess whether ADMolOrgGPT can generate molecules that simultaneously target two protein pockets through a direct RL-based optimization strategy in a single generative process. We present the pairwise optimization results for DYRK1A, BuChE and BACE-1 using the two-pocket reward function from Section Reward Modeling.

Figure 5 illustrates that, for all protein pairs studied, the model enhances the generation of molecules with favorable docking scores. After only a few epochs of training (epoch 15 for the first two cases, and epoch 14 for the third as it performed slightly better than epoch 15), the generated molecules showed improved docking scores for both protein pockets, as seen in Figure 5a (DYRK1A vs BuChE), Figure 5b (DYRK1A vs BACE-1), and Figure 5c (BuChE vs BACE-1). This is evident from the comparison between the blue boxplot (molecules without optimization) and the orange one (molecules after optimization). The observed enhancements ranged from 3% to 9%, which represent a meaningful improvement in docking performance.

**Figure 5:**
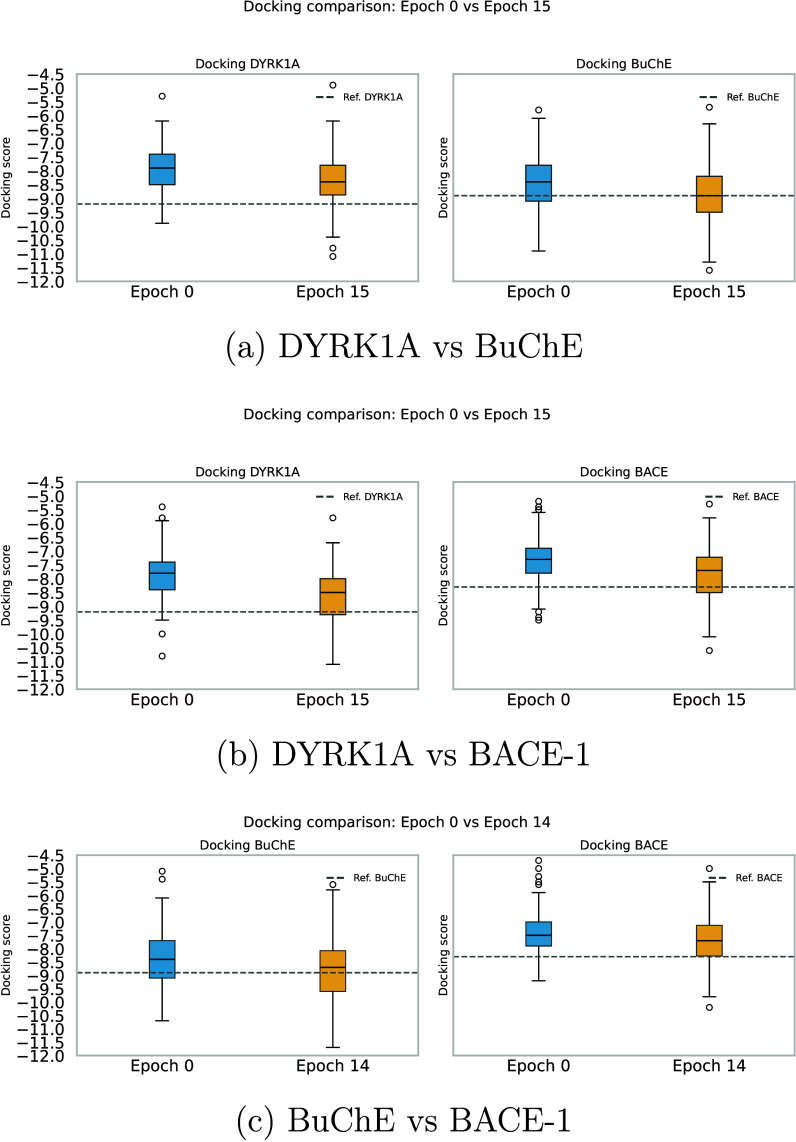
Multitarget docking optimization across three protein pairs.

To assess whether our model generates genuinely new dual-active molecules, rather than combinations of known scaffolds, we applied the following procedure:

- We collected all active molecules for DYRK1A and BuChE from ChEMBL.^90^
- We computed the Tanimoto similarity^77^ between the Morgan fingerprints of the ADMolOrgGPT generated molecules and those in the ChEMBL sets.

Each generated molecule was labeled as novel or not based on a maximum similarity threshold of 0.4.^91^ We then checked whether its scaffold was already present in ChEMBL. Table 5 shows that almost all generated molecules are structurally novel, and even those with higher similarity still contain unseen scaffolds.

**Table 5:** Confusion Matrix between Molecule and Scaffold Novelty

	scaffold novel	scaffold not novel
Novel molecule	241	5
Not novel molecule	10	0

Finally, Figure 6 summarizes representative outputs from ADMolOrgGPT under three generation settings for the three protein pockets. All the molecules represented meet the pharmacological property filters specified in Table 4.

**Figure 6:**
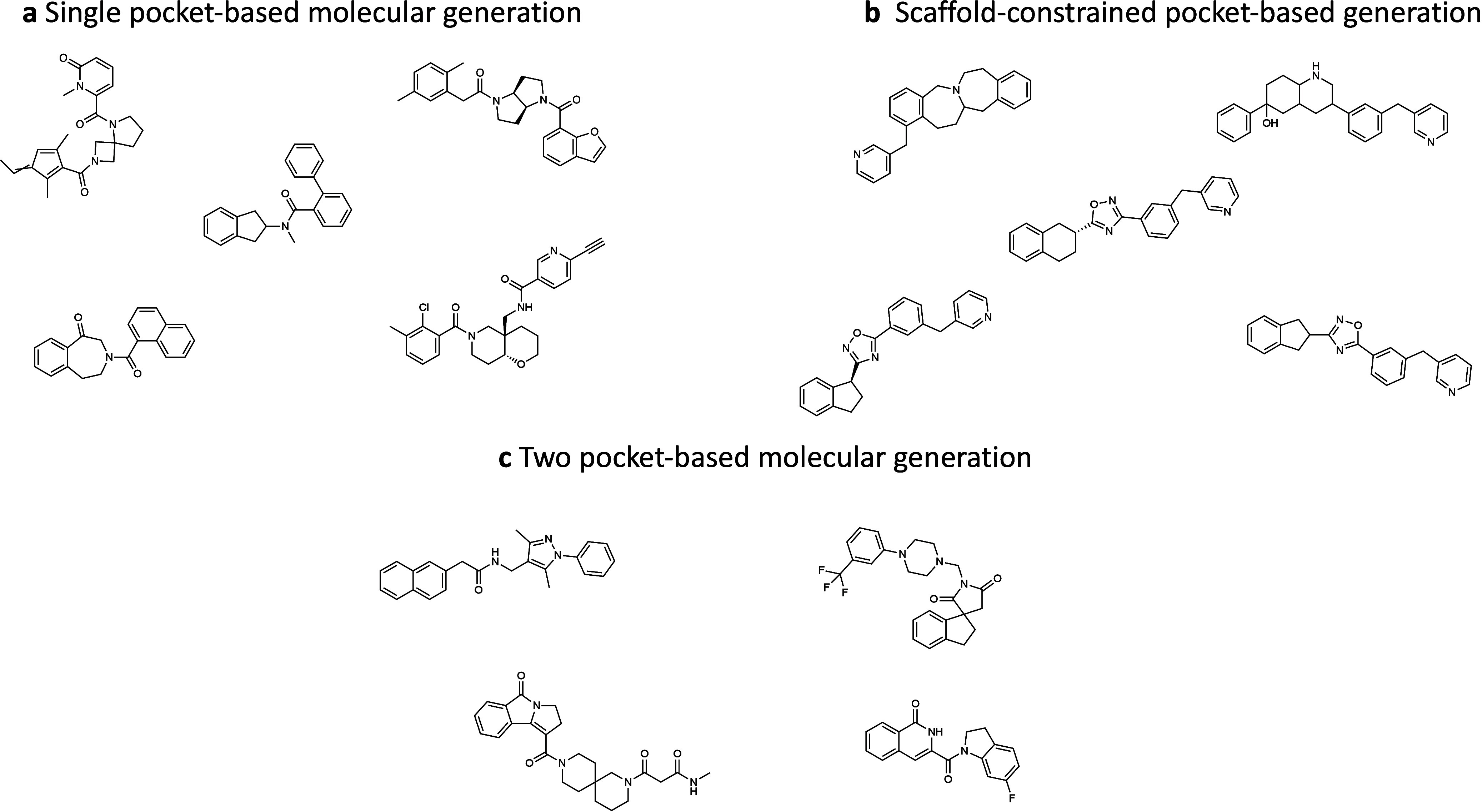
Representative molecules generated by ADMolOrgGPT across different tasks: (a) single pocket-based molecular generation; (b) scaffold-constrained pocket-based generation; (c) two pocket-based molecular generation.

### Docking Benchmark

To validate the molecules generated through the proposed generative workflow, a molecular docking analysis was carried out using the Schrödinger suite,^64^ following the methodology detailed in Subsection Docking Score Benchmarking. The results, summarized in Tables S4–S6 of the Supporting Information, confirm that all newly generated molecules successfully dock into the binding sites of their respective protein targets.

Overall, the generated compounds exhibit docking scores comparable to those of the reference ligands, suggesting a similar or potentially improved binding affinity. The docking poses reveal that these molecules consistently produce key interactions with crucial active-site residues, many of which are also involved in binding with the crystallographic ligands.

Figure 7 shows that the proposed ligands fit into the pocket of the target molecules. Besides, some of the interacting sites obtained for the generated molecules coincide with those obtained for the reference ligands, specifically those present in the crystallized 6EIF, 7Q1P, and 5TOL protein structures (see Figure S1 in the Supporting Information). For instance, in the case of the DYRK1A protein, Phe238 and Leu241 tend to form aromatic π–π stacking interactions and hydrogen bonds with the ligand, respectively. In the case of the BuChE protein, Trp82 and Phe329 also form aromatic π–π stacking interactions with the ligand in most of the predicted poses. As could be expected, some new interactions which are not present in the reference protein structures are established in the poses adopted by the generated molecules, thus enabling the exploration of new binding sites. It can also be appreciated in Figure 7 that the model is effectively learning to produce molecules that bind to specific regions and functional groups of the proteins. For example, in the case of the top ranked multitarget molecules, they interact with some common residues of the BuChe and DYRK1A proteins. In the case of BuChe protein, Phe329, Tyr332, Gly116, and Gly117 residues show common interactions for different molecules, while for the case of the DYRK1A protein this happens with Asn244 and Lys167 residues.

**Figure 7:**
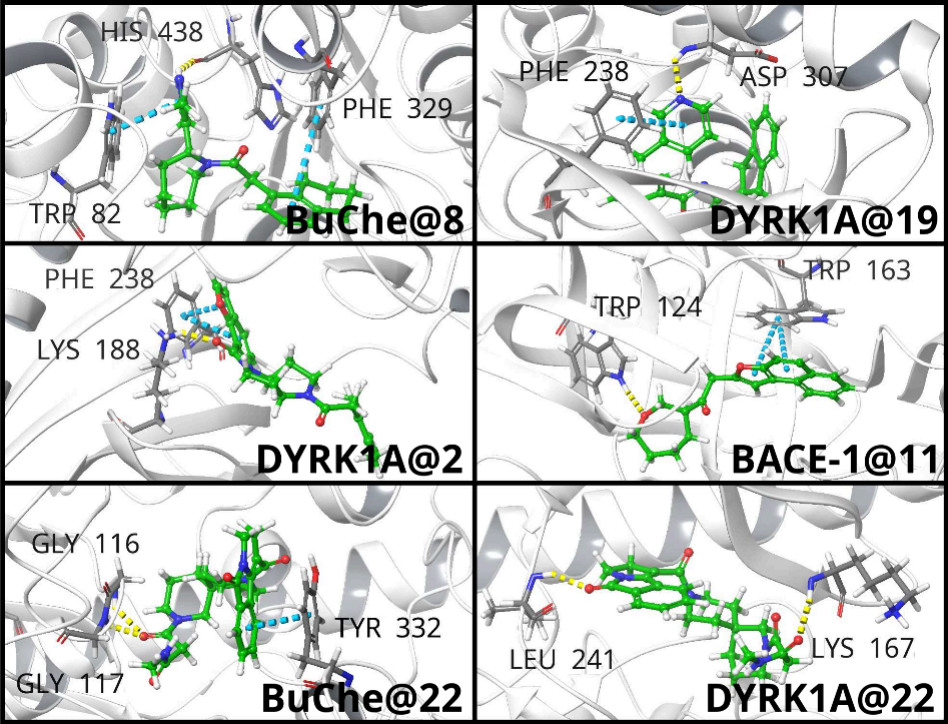
Schrödinger suite representation of docking poses adopted by some of the generated molecules.

In particular, the multitarget ligand @22, designed to inhibit both DYRK1A and BuChE, exhibited favorable docking scores against both targets, suggesting high predicted binding affinities. Moreover, @22 shows key interactions, such as hydrogen bonding with Leu241 and Lys167 in DYRK1A, and the π–π stacking with Trp332 and hydrogen bonds with Gly116 and Gly117 in BuChE. Additionally, @22 established novel contacts absent in the crystallized complexes, potentially stabilizing its binding and enabling engagement with alternative subpockets, which may contribute to its dual inhibitory capacity.

Taken together, these findings highlight the strong binding potential of the designed ligands, including both those with selectivity toward a single target and multitarget candidates such as @22. In particular, @22 stands out for its ability to engage essential residues in both enzymes while also exploring new interaction possibilities that could enhance its efficacy and selectivity.

## Discussion

We have showcased how our framework provides an innovative approach to address major limitations found in current protein pocket-based molecular generative models: their tendency to generate unrealistic or toxic molecules, their inability to optimize around a predefined scaffold, and their challenge when aiming for multitarget compounds capable of simultaneously addressing multiple protein pockets.

Unlike other models that depend on databases optimized for affinity scores, ADMolOrgGPT achieves results that are nearly on par with state-of-the-art methods like Pocket2Mol and DrugGPT, excelling in producing drug-like, nontoxic molecules with favorable LogP values. This demonstrates the effectiveness of our framework coupling transformer-based LLMs with RL in the domain of drug discovery.

We conclude by discussing relevant strategic aspects when implementing the framework, as well as some open issues.

### Analysis of Baseline Training Data Sets

We compared the physicochemical property distributions of the data sets used by the baseline models. Following the guidelines of TargetDiff,^92^ we extracted the molecules from CrossDocked and combined them with those from the jglaser/binding_affinity data set,^82^ forming the mixed baseline distribution used for analysis. For comparison, we randomly sampled approximately 600000 molecules from ZINC20 and from the mixed baseline distribution. Table 6 shows that the mixed baseline contains molecules with lower QED, higher LogP, greater synthetic difficulty, higher molecular weight, and a higher tendency to toxicity relative to ZINC20. For docking evaluation purposes, 256 molecules from each data set were docked against the three pockets under study.

**Table 6:** Comparison of Molecular Property Distributions between the Mixed Baseline Dataset and ZINC20^a^

	mixed baseline	ZINC20
Docking (kcal/mol, ↓)	–8.57 ± 1.56	–7.90 ± 1.04
QED (↑)	0.51 ± 0.21	0.74 ± 0.13
LogP (%, 0–5, ↑)	72.46 ± 0.06	99.99 ± 0.00
SAS (↓)	3.24 ± 0.93	3.18 ± 0.60
MW (↓)	438.31 ± 229.24	360.85 ± 54.46
Toxicity (%, ↓)	32.53 ± 0.06	6.48 ± 0.03

a ↑ Higher is better. ↓ Lower is better.

According to the Vina scoring function (eq 4), hydrophobic contacts and hydrogen bonding drive favorable docking scores. Consequently, Pocket2Mol and DrugGPT inherit a bias toward generating heavier and more hydrophobic molecules that naturally achieve better docking scores. In contrast, MolOrgGPT is pretrained on the filtered ZINC20 subset, which contains more drug-like molecules with moderate docking performance. From a drug-design perspective, this bias should be perceived beneficial rather than limiting. Early stage discovery pipelines apply physicochemical filtering prior to synthesis. Pretraining MolOrgGPT on ZINC20 therefore aligns with standard practice: the base model learns to generate drug-like structures, while PPO-RL subsequently optimizes binding without compromising more basic properties. Although MolOrgGPT does not incorporate structural protein information during pretraining, the PPO-RL stage effectively compensates for this, enabling the model to reach docking performances comparable to those of Pocket2Mol and DrugGPT.

### Model Comparison According to Size

As Figure 8 illustrates, and is generally assumed in the LLMs literature,^93^ increasing the number of parameters generally enhances performance. In our case, this means reducing the average docking scores of the generated molecules, making ADMolOrgGPT-XL the most relevant model. Interestingly, this model tends to converge in fewer epochs compared to its smaller variants, as Figure 9 shows. This faster convergence is important, as it suggests that the model can achieve high-quality solutions without deviating significantly from its original distribution.

**Figure 8:**
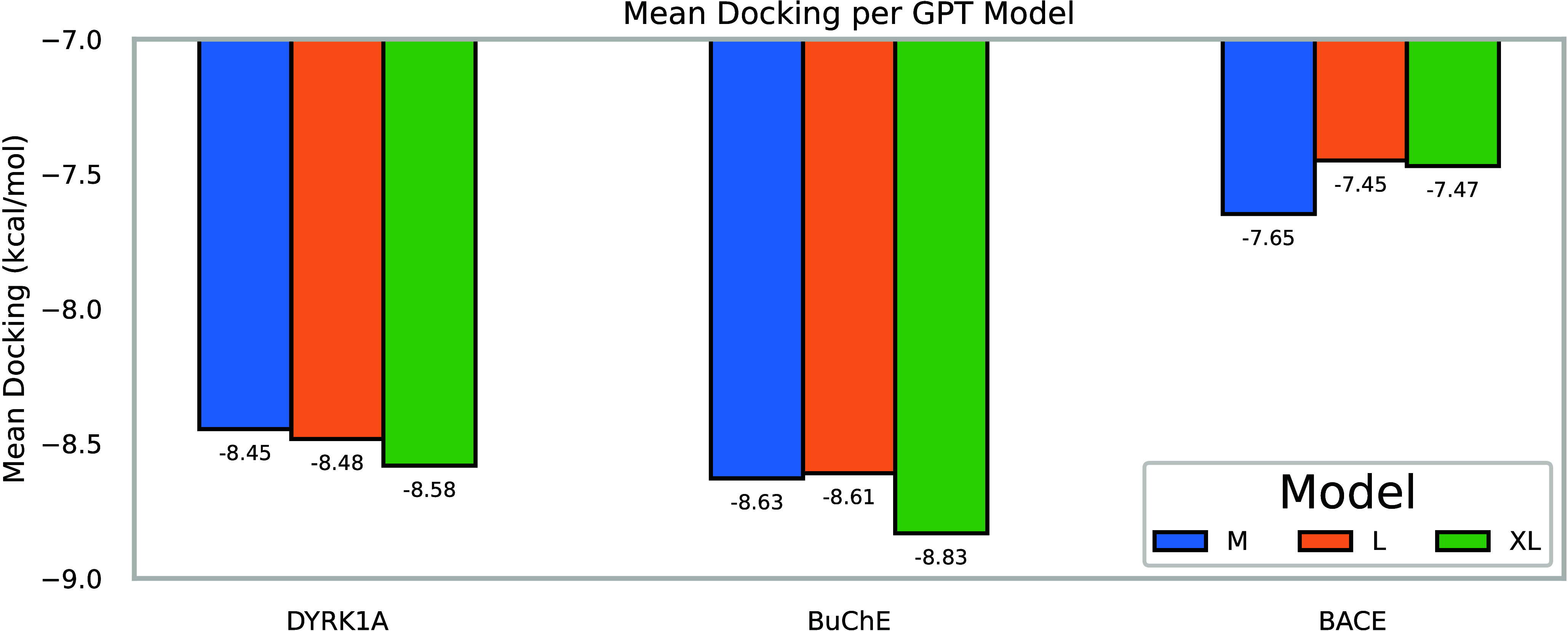
Docking results comparison according to model size

**Figure 9:**
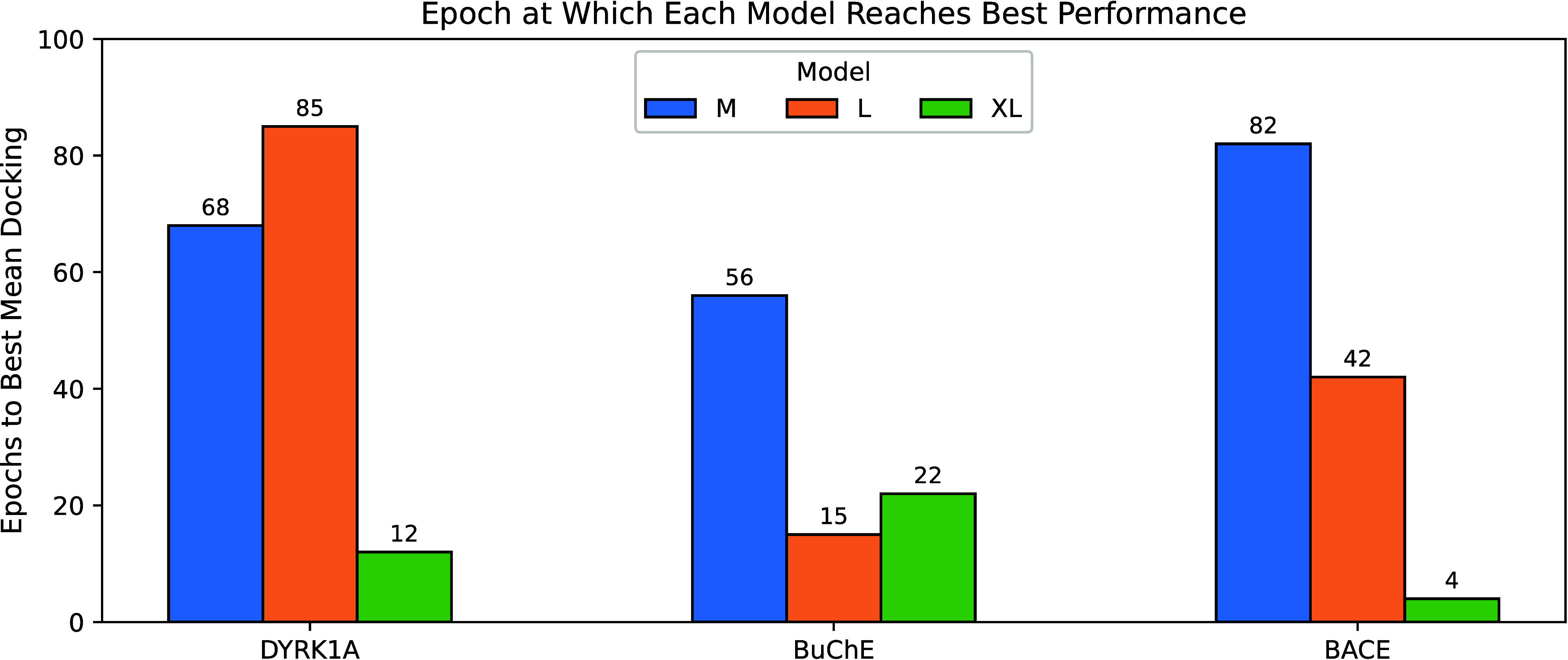
Best epoch comparison according to model size.

### Less Successful Attempts

While developing MolOrgGPT, we faced some less successful attempts that provide lessons for future related studies.

### Molecular Property Prediction

We began by assessing the performance of MolOrgGPT-M, L, and XL on different tasks related to molecular property prediction. For benchmarking purposes, we compared our models with the leading methods from the Triplet-Message Network (TRIMNET).^94^ The decision to use scaffold-based or random data splits was determined by the specific task at hand, and we adhered to the guidelines set forth by MoleculeNet.^95^ Overall, although our model performed strongly, it did not surpass current state-of-the-art methods. This outcome is consistent with prior reports^52^ showing similar trends, indicating that other architectures, such as Graph Neural Networks (GNNs), performed better.

### Tokenization

As discussed in the context of natural language processing,^96^ tokenization is key for the effective use of LLMs. The tokenizers initially considered were mentioned in Section Training Process. Recalling that the purpose of our study was to use RL to enhance pocket-based generation tasks, the use of a SMILES dictionary suffered inherent limitations, because when the algorithm varied its policy, many of the newly generated molecules were not chemically valid. As a result, the models did not improve in generating the property of interest. Importantly, using SELFIES mitigated this issue, as it always generates valid molecules.

### Multiple Properties Optimization

While our models can enhance the generation of molecules with specific properties, such as fixing a certain molecular weight, ensuring nontoxicity, or optimizing docking, they struggle when tasked with improving all of these characteristics simultaneously. Hence there is a need to further advance in adapting multiobjective RL methods and coupling them with LLM based molecular generation.

### PPO Hyperparameters Optimization

The PPO algorithm used is especially sensitive to the learning rate: if it is too high, it overshoots the solution, while if it is too low, the algorithm fails to effectively learn. Additionally, the number of epochs is a relevant factor in training. The ADMolOrgGPT-XL model requires fewer epochs to achieve an optimal solution, when compared with ADMolOrgGPT-M and L models. Moreover, if the model updates its weights for too many epochs, it risks reducing the diversity of the generated molecules, potentially converging to a single solution.

### Future Lines

From this research, several avenues emerge for future investigation. There are other RL methods that apparently can effectively fine-tune the LLMs, such as Augmented Hill Climb or Regularized Maximum Likelihood Estimation.^28,97^ Besides, the application of low-rank adaptation (LoRA)^98^ enables foundational models like MolOrgGPT to be efficiently fine-tuned using molecules that show high activity toward a specific protein of interest; this methodology could allow the model to generate molecules with high activity values.

Regarding model size, as discussed in Section Model Comparison According to Size, increasing the number of model parameters tends to enhance performance. Currently, our computational resources enable us to train a GPT-2 XL model with 1.5 B parameters. However, training even larger models could improve performance.

Finally, although our group focuses on AD, making the proteins studied in this work directly relevant, a similar pipeline could be applied to any target protein as long as a binding-site structure is available.

## Supplementary Material



## Data Availability

The training data for the foundational models can be downloaded from ZINC20 https://zinc.docking.org/. Code and model weights of MolOrgGPT and ADMolOrgGPT will be made available upon request by contacting the corresponding author.

## Notes

Tables S1–S6: ADMolOrgGPT generated molecules and corresponding docking scores, including single-pocket, scaffold-constrained, and multitarget strategies; Figure S1: Ligand–pocket interactions for selected protein–ligand complexes (PDF)
