# Supplementary material for: MolOrgGPT: De Novo Generation via Large Language Models and Reinforcement Learning
Source: J Chem Inf Model. 2026 Jan 7;66(2):910–22. doi: 10.1021/acs.jcim.5c02400 (PMC12848977; doi:10.1021/acs.jcim.5c02400)

Supplementary Information

MolOrgGPT: De novo Generation via Large Language Models and Reinforcement Learning

Pablo Varas Pardo,[†,‡,¶] Oscar Toledano,[§] Guillermo Marcos-Ayuso,[†] David Quesada,[†] and Nuria E. Campillo[*,§]

†AItenea Biotech S.L., 28014 Madrid, Spain
‡Instituto de Ciencias Matemáticas (ICMAT-CSIC), 28049 Madrid, Spain
¶Universidad Autónoma de Madrid, Escuela de Doctorado, Madrid, Spain
§Centro de Investigaciones Biológicas Margarita Salas (CIB Margarita Salas–CSIC), 28040, Madrid, Spain

Table of Content:

Tables S1-S3: ADMolOrgGPT-generated de novo molecules

Tables S4-S6: Docking score of the de novo molecules

Figure S1. Schrödinger suite representation of docking poses adopted by reference compounds

Table S1. Molecules generated with ADMolOrgGPT for each target (single pocket). The table reports the fitness value assigned by the model, along with the docking score obtained using AutoDock Vina. Additionally, molecular weight (MW), quantitative estimate of drug-likeness (QED), and synthetic accessibility score (SAS) are provided for each compound.

Target	*ID*	*SMILES*	*Fitness*	*Score*	*MW*	*SAS*	*QED*
DYRK1A							
	1	CC=C1C(C)=C(C(=O)N2CC3(CCCN3C(=O)C4=CC=CC(=O)N4C)C2)C(C)=C	0.99	-11.4	393.49	3.91	0.78
	2	CC1=CC=C(C)C(CC(=O)N2CC[C@H1]3[C@@H1]2CCN3C(=O)C4=CC=CC=5C=COC4=5)=C1	0.97	-10.7	402.49	3.32	0.66
	3	O=C1CN(C(=O)C2=CC=CC3=CC=CC=C23)CCC4=CC=CC=C41	0.96	-10.6	315.37	1.95	0.69
	4	C#CC1=CC=C(C(=O)NC[C@@]23CCCO[C@@H1]2CCN(C(=O)C4=CC=CC(C)=C4Cl)C3)C=N1	0.95	-10.5	451.95	3.84	0.72
	5	CN(C(=O)C1=CC=CC=C1C2=CC=CC=C2)C3CC4=CC=CC=C4C3	0.95	-10.5	327.43	1.96	0.69
	6	COC(=O)[C@H1](C1=CC=CC=C1)N2CCOC3=CC=C4C=C3C2CC4	0.99	-11.3	323.39	3.95	0.81
BuChE							
	7	CC1=CC(Cl)=CC(C)=C1O[C@H1](C)C(=O)NC2=CC=C3C(=C2)CCCC3=O	0.99	-11.1	371.86	2.67	0.83
	8	CC1=N[NH1]C(C)=C1[C@H1]2CCCCCN2C(=O)CC3=CC=CC4=C3CCCC4	0.98	-10.9	365.52	3.06	0.86
	9	CC1=CC(N2CCCCCC2)=CC=C1NC(=O)C(=O)NCC3CC4=CC=CC=C4C3	0.97	-10.7	405.54	2.26	0.76
	10	C[C@@H1]1C=2[NH1]C3=CC=CC=C3C=2CCN1C(=O)CC4=CC=CC=C4[N+1](=O)[O-1]	0.96	-10.6	349.39	2.77	0.58
BACE-1							
	11	O=C(CC1=CC2=C(C=CC3=CC=CC=C32)O1)[C@H1]4CC[C@H1]COC4	0.6	-9.2	307.37	3.31	0.72
	12	CN1C=CC2=CC=CC(C(=O)N3CC[C@H1]4CO[C@@H1](CNC(=O)C5=NC=CC=6OC=CC5=6)[C@H1]4C3)=C21	0.55	-9.1	458.52	4	0.51

13	CN1C(=O)C2=CC=C(C3=NC(C(C)(C)C4=CC=C(OCC(C)C)C=C4)=NO3)C=C2C1=O	0.55	-9.1	419.48	2.55	0.55
14	COC1=CC=C2N=C(C3CC3)C=C(C(=O)N4CCC[C@H1]4CO)C2=C1	0.5	-9	326.4	2.69	0.94

Table S2. Molecules generated with ADMolOrgGPT targeting DYRK1A (scaffold strategy). The table reports the fitness value assigned by the model, along with the docking score obtained using AutoDock Vina. Additionally, molecular weight (MW), quantitative estimate of drug-likeness (QED), and synthetic accessibility score (SAS) are provided for each compound.

ID	*SMILES*	*Fitness*	*Score*	*MW*	*SAS*	*QED*
15	C1=CC=C(CC2=CC=CN=C2)C3=C1CN4CCC5=CC=CC=C5CC4CC3	0.99	-11.4	354.497	2.82	0.67
16	C1=CC=C(CC2=CC=CN=C2)C=C1C3CNC4CCC(O)(C5=CC=CC=C5)CC4C3	0.9	-10.6	398.55	3.73	0.61
17	C1=CC=C(CC2=CC=CN=C2)C=C1C3=NOC([C@@H1]4CCC5=CC=CC=C5C4)=N3	0.94	-10.4	367.452	2.76	0.51
18	C1=CC=C(CC2=CC=CN=C2)C=C1C3=NC([C@H1]4CCC5=CC=CC=C54)=NO3	0.92	-10.2	353.425	2.77	0.53
19	C1=CC=C(CC2=CC=CN=C2)C=C1C3=NC(C4CC5=CC=CC=C5C4)=NO3	0.90	-10.1	353.425	2.34	0.54

Table S3. Molecules generated with ADMolOrgGPT targeting DYRK1A and BuChE (doble-pocket). The table reports the fitness value assigned by the model, along with the docking score obtained using AutoDock Vina. Additionally, molecular weight (MW), quantitative estimate of drug-likeness (QED), and synthetic accessibility score (SAS) are provided for each compound.

ID (publ)	*SMILES*	*Fitness*	*Score DYRK1A*	*Score BuChE*	*MW*	*SAS*	*QED*
20	CC1=NN(C2=CC=CC=C2)C(C)=C1CNC(=O)CC3=CC=C4C=CC=CC4=C3	0.95	-10.2	-11.1	369.468	1.99	0.56
21	O=C1CC2(CCC3=CC=CC=C32)C(=O)N1CN4CCN(C5=CC=CC(C(F)(F)F)=C5)CC	0.95	-10.1	-11.6	369.468	3.46	0.68

22	CNC(=O)CC(=O)N1CCCC2(CCN(C(=O)C3=C4C5=CC=CC=C5C(=O)N4CC3)CC2)C1	0.93	-10.8	-10	450.539	3.46	0.718
23	O=C(C1=CC2=CC=CC=C2C(=O)[NH1]1)N3CCC4=CC=C(F)C=C43	0.93	-10.1	-10.5	308.312	2.19	0.75

Table S4: Docking scores of the generated molecules using the single-pocket methodology. Their average values and standard deviations are also included.

Target	*Molecule ID*	*Score*
DYRK1A		
	XMD7-117*	-9.54
	2	-7.91
	3	-7.75
	4	-7.41
	5	-7.01
	1	-6.59
	Average	-7.3±0.2
BuChE		
	8UW*	-6.57
	8	-10.47
	7	-8.62
	6	-7.40
	10	-6.82
	9	-6.48
	Average	-8.0±1.5
BACE-1		
	7H3*	-6.03
	11	-6.18
	12	-5.78
	14	-5.71
	13	-4.42
	Average	-5.5±0.7

* reference compounds

Table S5: Docking scores of the generated molecules using the scaffold methodology targeting DYRK1A. Their average values and standard deviations are also included.

Molecule ID	*Score*
XMD7-117*	-9.54
19	-9.14
18	-8.90
16	-8.65
17	-7.14
15	-4.65
Average	-7.7±1.7

* reference compounds

Table S6: Docking scores of the multitarget generated molecules (double-pocket methodology) targeting DYRK1A and BuChE. Their average values and standard deviations are also included.

Molecule ID	*Score (DYRK1A)*	*Score (BuChE)*
23	-8.73	-4.59
22	-7.98	-7.08
20	-6.30	-9.50
21	-4.83	-7.79
Average	-7.0±1.5	-7.2±1.8

Figure S1. Schrödinger suite representation of docking poses adopted by reference compounds

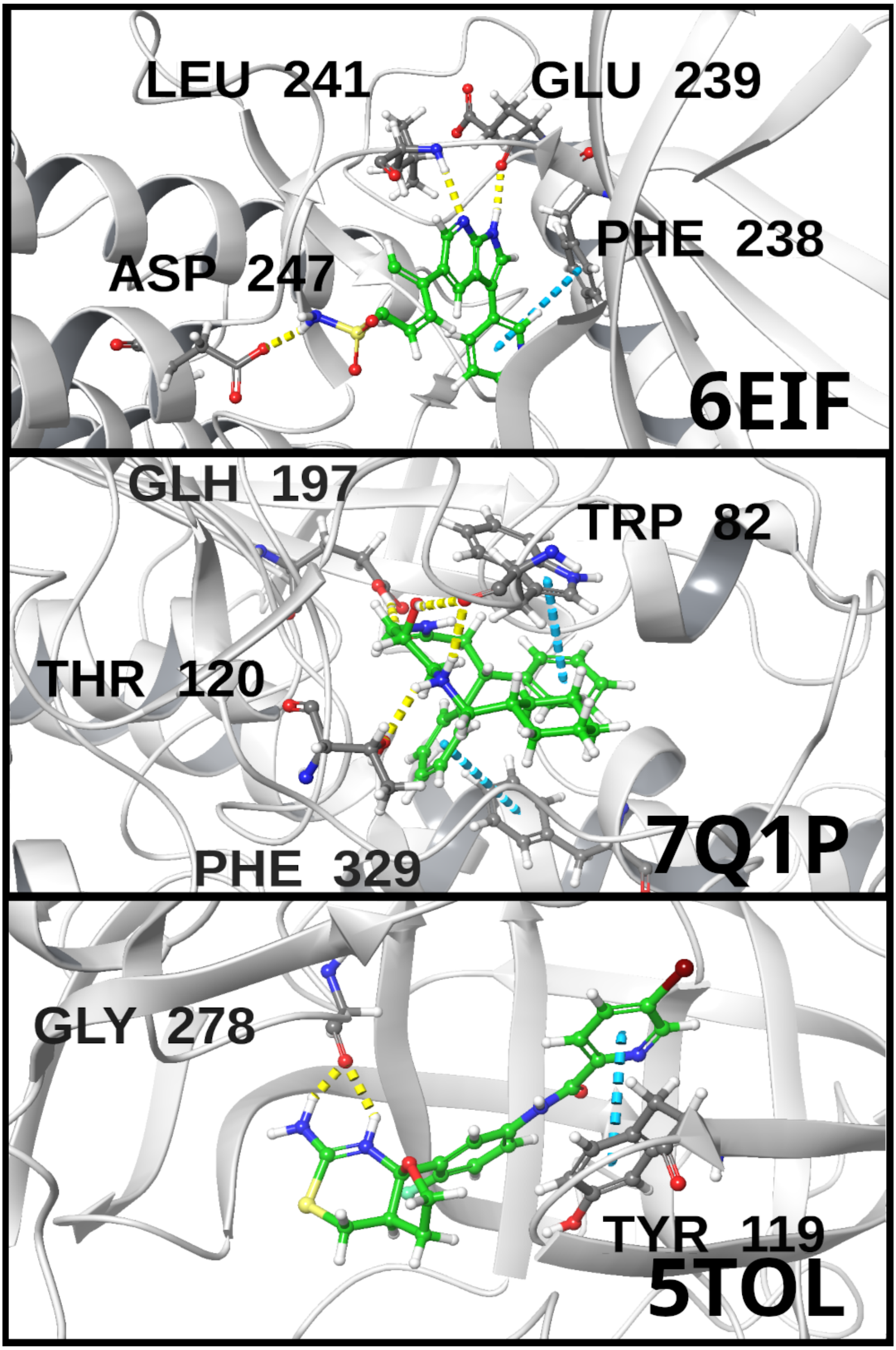

Supplement: Supplementary file 1 [file ci5c02400_si_001.pdf]
